# Mechanism Influencing Older People’s Willingness to Use Intelligent Aged-Care Products

**DOI:** 10.3390/healthcare9070864

**Published:** 2021-07-08

**Authors:** Biao Wang, Rui Zhang, Ying Wang

**Affiliations:** 1 University of Science and Technology of China, Hefei 230026, China; biaowang2021@163.com; 2 Hefei University of Technology, Hefei 230009, China; 2018110723@mail.hfut.edu.cn

**Keywords:** intelligent aged-care products, perceived quality, emotional attachment, willingness to use

## Abstract

Background: With the continuous integration of information technology in the aged-care industry, intelligent aged-care products have gradually appeared, positively promoting the development of the industry. To support the use of these products and to help older people to improve their own health literacy, we built a theoretical model of the mechanism influencing older people’s willingness to use intelligent aged-care products. Methods: A total of 241 valid questionnaires were collected through surveys in aged-care institutions in Anhui Province, China, for empirical analysis. Results: Older people’s perception of quality can significantly affect their emotional attachment and willingness to use these products. Emotional attachment has a significant positive impact on the willingness to use. Self-perceived ageing can also significantly affect the emotional attachment and willingness of older people to use these products. Conclusion: Through empirical analysis, the comprehensive mechanism influencing older people on the willingness to use intelligent aged-care products is clarified, which can help older people to better deal with the problems caused by ageing and help aged-care institutions better relieve the pressure on nursing staff.

## 1. Introduction

In the 21st century, China is facing the following two major trends: an increase in the number of older people and a wave of technology [1]. According to the 2019 National Economic and Social Development Statistical Bulletin issued by the National Bureau of Statistics, China’s population is ageing rapidly. As of the end of 2019, the total population of mainland China was 1400.05 million, of which 175.99 million are 65 years old and above, accounting for 12.57% of the total population. By 2050, China will enter a stage of further ageing [2]. With an increase in age, older people tend to be more concerned about their own health problems and will take the initiative to take certain measures conducive to promoting health to alleviate the negative effects of ageing. Therefore, effective means are needed to solve a series of social problems caused by the ageing population [3].

Technology has the potential to help elderly monitor and maintain health and manage health conditions and diseases. The previous literature has shown that elderly people are fraught with apprehension when using healthcare information technology, which may hinder technology’s acceptance and adoption [4]. However, Mitzner et al. found in their study that elderly people have more positive than negative attitudes towards the technology they use, which contradicts the stereotype that elderly people are afraid or unwilling to use technology [5]. Influenced by the concept of merging artificial intelligence and medical treatment, intelligent aged-care products are continuing to appear in major aged-care organizations; here, we define intelligent elderly care products as intelligent auxiliary products that can help older people actively cope with ageing, including intelligent sleep detectors, intelligent monitoring, intelligent mattresses, positioning bracelets, intelligent robots, and other intelligent devices. With the continuous development of information technology, the degree of informatization of the aged-care industry is also increasing and intelligent elderly care products are gradually becoming an effective means to help aged-care organizations improve nursing efficiency and improve the physical and mental health of older people. For example, Portugal et al. showed that the use of intelligent aged-care products helps users in understanding their own health status and improves their own health literacy [6]; Xie et al. reported that the use of intelligent aged-care service products can help alleviate the nursing pressure in aged-care organizations, thereby alleviating the problem of the high turnover rate of nursing staff [7] and achieving a high quality of care [8].

Previous scholars have discussed the design process of intelligent aged-care products, but research is lacking on the willingness of older people to use these products [9]. As the most important audience of intelligent aged-care products, older people’s willingness to use the products plays an important role in the effective use of the products [10]. From a rational point of view, the success model of an information system should well-explain an individual’s subsequent use or use intention of and satisfaction with a certain information system or product [11,12]. Considering the perceptual characteristics of older people, their emotional attachment to intelligent aged-care products can significantly affect the final use intention [13].

Therefore, in this information society environment, the mechanism influencing older people’s willingness to use intelligent aged-care products needs further exploration. In this research, we focused on the willingness of older people to use these products in terms of the following two questions:

(1) What factors affect the emotional attachment of older people to intelligent aged-care products?
(2) What is the comprehensive mechanism through which the perceived product quality, emotional attachment, and self-perception of ageing affect the willingness of older people to use intelligent aged-care products?

The rest of the study is organized as follows: Section 2 presents the theoretical basis of the study and the hypotheses. Section 3 introduces the research and data analysis methods. Section 4 describes the data analysis. Finally, the results and findings are discussed, and the theoretical and practical contributions are described.

## 2. Background

In this part, we introduce the literature review and hypotheses. The literature review introduces the concepts and research status of information system success models, attachment theory, and willingness to use in detail. Six hypotheses are proposed for the theoretical framework of this study.

### 2.1. Literature Review

#### 2.1.1. Willingness to Use

The term willingness originated in marketing theory and was mainly used to study consumers’ purchase intention, that is, the probability of consumers being willing to purchase a certain product or service [14]. Later, some scholars introduced willingness into the context of information technology acceptance and adoption to study the connotation of willingness to use [15]. Willingness to use refers to a user’s intention to use the product or service and may also refer to the psychological tendency of a user to produce a certain behavior [16]. Moreover, other previous studies have found that the stronger an individual’s willingness to perform a certain behavior, the greater the possibility of performing the behavior [10,17]. In this paper, we believe that the willingness of older people in elderly care institutions to use intelligent elderly care service products refers to a psychological tendency of older people to use such products [16], and this psychological tendency will have a high probability of causing a specific use behavior to occur.

#### 2.1.2. Information System Success Model

The information system success model was first proposed by DeLone and McLean in 1992 [18]; they further improved and updated the model based on the evaluation of other scholars’ contributions to the model, and finally constructed a mature information system success model [19]. The updated model consists of the following six interrelated dimensions of information system success: information quality, system quality, service quality, use and intention to use, user satisfaction, and net benefits [19,20]. In the information system model, DeLone and McLean stated that the quality of the system can be evaluated from the following three aspects: information quality, system quality, and service quality [19,20]. Among these aspects, information quality is a key variable used to measure semantic success, reflecting the completeness, accuracy, and real-time degree of the information output by the system or product during use [21]. System quality is a key variable used to measure technical success, reflecting the reliability and efficiency of the system’s or product’s processing of its own software and data analysis during use [22]. Service quality is also a key variable for measuring semantic success [23,24], reflecting the credibility, timeliness, personalization, and specialization of the system or product in use [22]. Many previous studies have verified the effectiveness of this model [25,26,27], showing that these three dimensions are one of the key factors that determine the success of an information system or product [20]. These factors also have a certain impact on the individual’s emotions and behavior.

#### 2.1.3. Attachment Theory

The concept of attachment was first proposed by Bowlby, who initially stated that attachment refers to the strong emotional connection between a baby and their mother [28]. Subsequently, many scholars conducted research on this basis, but most of them still defined attachment as a strong and lasting emotional connection between an individual and a specific individual, emphasizing the emotional connection between people [29,30]. Subsequently, Schults applied attachment theory to the marketing field for the first time and extended it to the context of the emotional connection between people and things, producing a breakthrough in attachment theory [31]. On this basis, later scholars conducted in-depth research and found that individuals form attachments to places [32], products, and brands [33], and that this attachment relationship is an important driving force that promotes individual commitment, loyalty, and other behavioral will.

Emotional attachment has been developed on the basis of attachment theory. Wang and Fesenmaier stated that emotional attachment refers to the personal satisfaction of the self through possessions, and the formation of a connection with the self, thereby forming a strong emotional connection to possessions, places, or brands [34]. Compared to other age groups, older people are more likely to have an emotional attachment to certain trusted items due to certain characteristics common among elderly people, such as reduced physical ability or memory loss; the stronger the emotional attachment is, the stronger their motivation to devote personal resources to using the product is [35].

### 2.2. Model Construction and Hypothsis

#### 2.2.1. Product Quality Perception and Willingness to Use

The information system success model shows that the individual’s perception of the information, system, and service qualities of a system or product will significantly impact user satisfaction and behavior intentions [19]. Previous research on the success model of information systems focused more on the application of a certain information system or online software to determine the success of the construction of this information system or platform [20,26,36]. For example, in their research on the government office environment, Mardiana et al. found that system quality and service quality are important factors affecting the willingness of government staff to use information systems [11]. Lee et al. found, in a study of application service provider (ASP) software usage intention, that information quality has a significant positive effect on improving user satisfaction, trust, and usage intention [37].

However, with the continuous development of information technology, the use of intelligent products has gradually become a trend. In the process of using intelligent products, the degree of an individual’s perception of the quality of the intelligent products can affect the individual’s internal emotions and behavioral wishes [12,38,39]. Similarly, we argue that in aged-care organizations, the perception of the quality of intelligent service products by older people will also affect their inherent willingness to use the products [12]. When the product information quality and system quality are perceived to be high, the trust of older people in the intelligent products increases [40]; when the perceived quality of products and services is high, the satisfaction of older people with the intelligent products increases [41]. Both of these factors in turn promote the inherent willingness of older people to use the products. Based on the above, the following hypotheses are proposed:

**Hypothesis** **1a** **(H1a).** *Older people’s perceived information quality of intelligent aged-care products has a significant positive impact on their willingness to use those products*.

**Hypothesis** **1b** **(H1b).** *Older people’s perceived system quality of intelligent aged-care products has a significant positive impact on their willingness to use these products*.

**Hypothesis** **1c** **(H1c).** *Older people’s perceived service quality of intelligent aged-care products has a significant positive impact on their willingness to use those products*.

#### 2.2.2. Product Quality Perception and Emotional Attachment

Individuals’ perception of product quality can significantly affect their internal emotional response. When individuals have a high perception of product quality, a positive emotional response is promoted [42]. Research by Hwang et al. also showed that product or service quality is an important indicator for measuring user emotional response [43]. Emotional attachment, as an important variable of emotional reflection, refers to individuals satisfying their own needs through possessions and forming a connection with the self, thus forming a strong emotional connection to possessions, places, or brands [34].

With the continuous development of intelligent products, individuals’ perception of the quality of intelligent products has gradually become an important factor in judging whether it will meet their needs. When an individual perceives that a particular product is of high quality, the feelings of trust and satisfaction in the product are more easily promoted [41] and, ultimately, specific behavior intentions are generated [44]. This is especially true for older people: when they perceive that intelligent aged-care products are of high quality and can meet their own health needs, they will have an emotional connection to the products, and then form an emotional attachment. Based on the above, the following hypotheses are proposed:

**Hypothesis** **2a** **(H2a).** *Older people’s perceived information quality of intelligent aged-care products has a significant positive impact on their emotional attachment to the products*.

**Hypothesis** **2b** **(H2b).** *Older people’s perceived system quality of intelligent aged-care products has a significant positive impact on their emotional attachment to the products*.

**Hypothesis** **2c** **(H2c).** *Older people’s perceived service quality of intelligent aged-care products has a significant positive impact on their emotional attachment to the products*.

#### 2.2.3. Self-Perception of Ageing, and Emotional Attachment and Willingness to Use

Self-perception of ageing refers to the part formed after the individual internalizes the negative or positive attitudes of others toward oneself, that is, the general attitude of others towards oneself. After Mead’s study, some scholars applied self-perception variables to the perception of the degree of self-ageing of older people and proposed the concept of self-perception of ageing. Barker stated that self-perception of ageing refers to the subjective perception and emotional response of older people when they are threatened by physical, psychological, and social ageing. This perception and response affect the behavioral tendency of older people in the ageing process [45].

Many previous studies have been conducted on the self-perception of ageing. They have found that when older people are satisfied with their self-perception, and see themselves as active or healthy, the self-perception more effectively improves the actual physiology [46] and mental health level [47]. Langer and Rodin found that elderly people with a positive self-perception of ageing will be more active and responsible and will have an emotional connection with products and measures that promote their own health, which then enhances the emotional attachment to the product, meeting their needs for maintaining a good and healthy lifestyle [48]. Similarly, when individuals’ self-perceived ageing degree is relatively positive, the increase in older people’s willingness to use intelligent aged-care products is promoted. For example, Yahui and Yanhua showed that the more positive the self-perception of ageing of older people is, the stronger their willingness to take measures to help their own health status is [49] and intelligent aged-care products are an important and effective means to help older people improve their health status [50]. For elderly people with a positive self-perception of ageing, their willingness to use intelligent aged-care products will be stronger.

Therefore, we concluded that as older people’s self-perception of ageing becomes more active, the use of intelligent aged-care products will increase. Relationships with attachment will also increase the willingness to use the product. Based on the above, the following hypotheses are proposed:

**Hypothesis** **3** **(H3).** *The self-perception of ageing of older people has a significant positive impact on the emotional attachment to intelligent aged-care products*.

**Hypothesis** **4** **(H4).** *The self-perception of ageing of older people has a significant positive impact on their willingness to use intelligent aged-care products*.

#### 2.2.4. Emotional Attachment and Willingness to Use

According to the theory of self-inflation [51], emotional attachment refers to a strong relationship between people or people and things, which can produce strong motivation and behavioral results [51]. When an individual is strongly attached to a specific goal, they will be committed to that goal and will work hard to maintain interaction with the goal, and may spend more time, energy, and financial resources to strengthen this relationship [52] and then strengthen the connection between themselves and other things [53]. Previous studies have discussed the emotional attachments of individuals to objects, brands, virtual communities, places, and possessions from different research backgrounds, finding that these emotional attachments impact consumers’ purchase intentions and behaviors [51,54,55], brand loyalty [56,57], online community participation intention [58], and product use intention [13].

We concluded that, in aged-care organizations, the emotional attachment of older people to intelligent aged-care products refers to a strong emotional connection between older people and the products. This strong emotion can significantly promote their willingness to use the products. When older people are more emotionally attached to a product, their willingness to use it will also be stronger. Based on the above, the following hypothesis is proposed:

**Hypothesis** **5** **(H5).** *The emotional attachment of older people to intelligent aged-care products has a significant positive impact on their willingness to use the product*.

#### 2.2.5. The Moderating Effect of Self-Perceived Ageing on the Relationship between Perceived Product Quality and Emotional Attachment

Self-perceived ageing, as an important individual characteristic of older people, has a certain impact on the relationship between an individual’s perception of external product quality and emotional attachment. For older people, when they have a more self-perception of ageing, it will promote them to take active coping measures in the ageing process [45], which is conducive to physical and mental health [59]. Furthermore, the perception of information, system, and service quality of intelligent aged-care products will be better. A relatively positive self-perception of ageing will also increase the emotional attachment degree of older people to products that meet their needs for maintaining a good and healthy lifestyle [48], and the overall degree of emotional attachment to intelligent aged-care products will also be high. Based on the above, the following hypotheses are proposed:

**Hypothesis** **6a** **(H6a).** *A moderating effect of self-perceived ageing on the relationship between perceived information quality and emotional attachment exists*.

**Hypothesis** **6b** **(H6b).** *A moderating effect of self-perceived ageing on the relationship between perceived system quality and emotional attachment exists*.

**Hypothesis** **6c** **(H6c).** *A moderating effect of self-perceived ageing on the relationship between perceived service quality and emotional attachment exists*.

**Figure 1:**
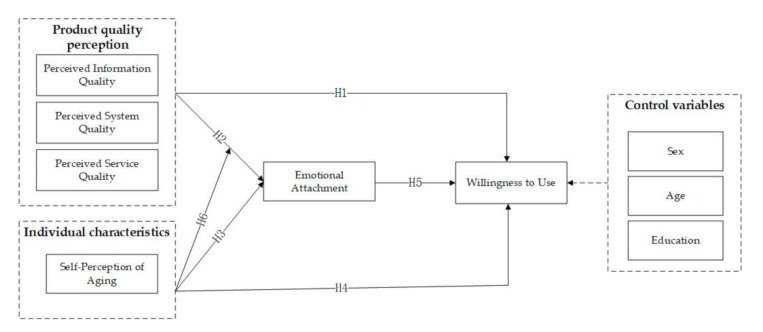
Theoretical model diagram.

The model is shown in Figure 1.

## 3. Methods

We mainly collected data through questionnaires. Firstly, a questionnaire was designed according to the mature scale. Secondly, the questionnaire was administered to older people in large nursing institutions in Anhui Province, China. Finally, SPSS (SPSS Inc., Chicago, IL, USA) and SmartPLS software (Bönningstedt, Germany) were used to analyze the collected effective data.

### 3.1. Measurement

The main latent variables of this research model are perceived information quality (PIQ), perceived system quality (PSYQ), perceived service quality (PSEQ), self-perception of ageing (SPA), emotional attachment (EA), and willingness to use (WTU). The measurement indicators of the six latent variables are shown in Table 1. The questionnaire adopted a 7-point Likert scale, with numbers from 1 to 7 indicating the degree of approval of the respondent for the question, where 1 represents strong disapproval and 7 represents strong approval.

### 3.2. Survey Objects and Data Collection

Considering that this paper is a study on the willingness of elderly people to use intelligent aged-care products in aged-care institutions, older people will be more able and willing to use intelligent aged-care products, which can give full play to the intrinsic value of intelligent pension products and better serve older people. At the same time, by referring to the research of Kuoppamaki et al. [66], the research object of this paper is mainly an older age group over 55 years old in aged-care institutions, to explore the influence mechanism model of their willingness to use intelligent aged-care products.

We focused on older people in aged-care organizations in Anhui Province. First, to verify the validity of the questionnaire, we administered 50 pre-survey questionnaires, and revised the questionnaire according to the survey results. Subsequently, the revised questionnaire was released to some aged-care organizations with intelligent aged-care products in Anhui Province. A total of 300 questionnaires were issued, and 241 valid questionnaires were recovered, for an effective rate of 80.33%. The collection of the questionnaire is shown in Table 2.

In general, the number of men and women in this survey was relatively equal; in terms of age, the population over 65 years old accounted for 68.88%. In terms of education level, high school/technical secondary school and below accounted for 53.53%. Among the marital status, the married elderly accounted for 47.72%. Finally, in terms of monthly income, the monthly income was mainly below 4500-yuan, accounting for 71.37% of the population.

## 4. Results

This section mainly describes the process of data analysis and processing. Based on the valid returned samples, firstly, descriptive statistics were applied to the questionnaire data, followed by reliability and validity testing, model fitting and hypothesis testing, and finally a conclusion was drawn.

### 4.1. Measurement Model

#### 4.1.1. Descriptive Statistical Analysis

First, the descriptive statistics were calculated, and the statistical results are shown in Table 3. The average value of the measurement problem reflects the fluctuation in the collected data, which ranged from 4.365 to 4.867, with less fluctuation and more concentrated data. The factor loading of each item is higher than 0.7, which indicates that the correlation between test items and measurement variables is very high, and the item setting is reasonable.

#### 4.1.2. Reliability and Validity Tests

Next, to ensure the reliability and validity of the questionnaire, the fitting degree of the model, and the validity of the hypothesis test, we used SmartPls 3.0 (Bönningstedt, Germany) to test the reliability and validity of the measurement variables, mainly testing the following parameters: the reliability and internal consistency of each item, and the discriminant validity and convergence of the model. These parameters can reflect the quality of model construction. In the reliability test, Cronbach’s alpha reflects the reliability of each item in the questionnaire. The higher the coefficient is, the higher the reliability of the scale is. The Cronbach’s alpha of all the variables in this questionnaire were above the recommended standard of 0.70 [67], ranging from 0.841 to 0.916, indicating that the scale selected in this study had high reliability.

Next were the validity tests of the questionnaire. First was the test of aggregation validity. Convergent validity is a test used to measure the consistency of multiple items of the same concept. Compound reliability (CR) and average variance extraction (AVE) are the effective indicators for testing aggregate validity [68]. The factor loading of each observation variable exceeded the recommended value of 0.6, which showed that the observed variables of the model had a high correlation with the structural variables to which they belong [69]. The CR value of this model ranged from 0.921 to 0.943, which exceeds the recommended value of 0.7, indicating that the internal consistency of the model variables is good. The AVE in the model was within the range of 0.736 and 0.847, which exceeds the recommended value of 0.5 [68], indicating that the observed variables in the model can explain each measurement dimension well [70].

The second validity test was the test of discriminant validity. Discriminant validity refers to the degree of irrelevance between the measurement index of each latent variable and the measurement indicators of other latent variables [71]. In this model, the square root of each observation variable AVE was greater than its correlation coefficient with other observation variables, which showed that each observation variable had a strong discriminant coefficient. The discrimination was very high [70]. The measurement indicators are shown in Table 4.

Through the above analysis, we concluded that the questionnaire design in this study was reasonable and had good reliability and validity. The evaluation structure of the measurement model was effective and reasonable, and further structural model fitting analysis could be performed.

### 4.2. Structural Model Analysis

**Figure 2:**
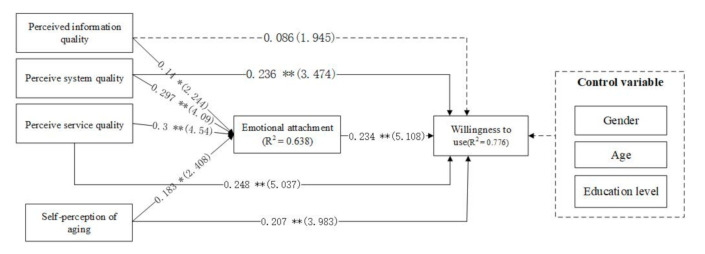
Model fit chart. Note: * *p* < 0.05; ** *p* < 0.01.

The structural model identifies causal relationships between the variables in the model [72]. In this study, the theoretical model proposed by SmartPLS3.0 software (Bönningstedt, Germany) was used for structural equation analysis. The detailed analysis results are shown in Figure 2.

The above model analysis results show the following: First, we analyzed the impact of product quality perception on the willingness to use of older people. Older people’s perceived system quality (β = 0.236, *p* < 0.01) and perceived service quality (β = 0.248, *p* < 0.01) of intelligent aged-care products had a significant positive impact on their willingness to use; therefore, H1b and H1c were supported. Older people’s perceived information quality (β = 0.086, *p* > 0.01) of intelligent aged-care products had no significant impact on their willingness to use; therefore, H1a was rejected.

Second, we analyzed the impact of product quality perception on the emotional attachment of older people. Older people’s perception of the information quality (β = 0.14, *p* < 0.05), the system quality (β = 0.297, *p* < 0.01), and the service quality (β = 0.3, *p* < 0.01) of intelligent aged-care products significantly impacted their emotional attachment to the products. Thus, H2 was supported.

Third, we analyzed the influence of the self-perception of ageing at the individual level on the emotional attachment and willingness to use intelligent aged-care products. Self-perceived ageing had a significant positive impact on emotional attachment (β = 0.183, *p* < 0.05), and the common explanation variance was 63.8%. H3 was, therefore, supported. Similarly, self-perceived ageing had a significant effect on willingness to use the products (β = 0.207, *p* < 0.01), also a significant positive effect. Thus, H4 was supported.

Finally, we analyzed the influence of the emotional attachment of older people to the intelligent aged-care products on their willingness to use these products. We found that the emotional attachment of older people to the product (β = 0.234, *p* < 0.01) had a significant positive impact on their willingness to use it, and the common explanation variance was 77.6%. H5 was, therefore, supported.

### 4.3. Moderation Effect

**Figure 3:**
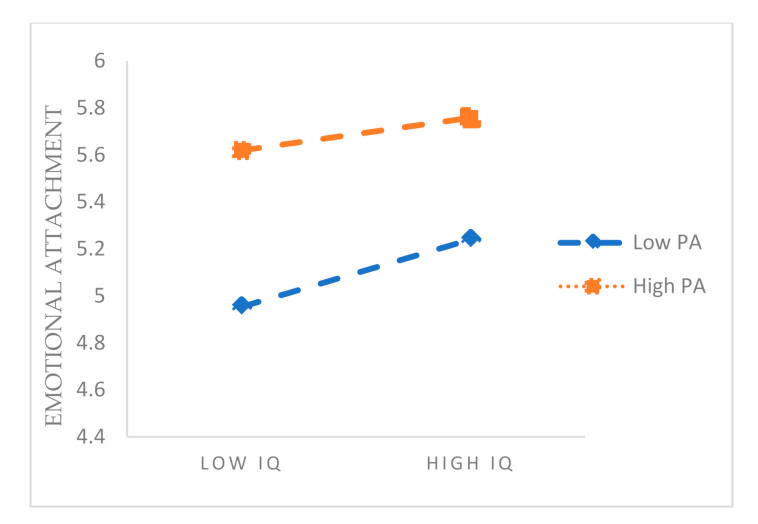
Moderating effect.

Then, we verified the moderating effect of the self-perception of ageing. SPSS (SPSS Inc., Chicago, IL, USA) was used to study the moderating effect of the degree of individually perceived ageing on the relationship between perceived information quality and emotional attachment. The results are shown in Table 5. The results show that when the interaction term was added to the model, the interaction term was also significant (β = −0.15, *p* < 0.01). Figure 3 shows that when the individual’s self-perception of ageing was low, there was a strong positive correlation between the perceived information quality and emotional attachment, whereas the overall emotional attachment was low. When the individual’s self-perception of ageing was high, the positive correlation between the perceived information quality and emotional attachment was weaker, and the overall emotional attachment was higher. Therefore, the self-perception of ageing plays a negative role in the adjustment of perceived information quality and emotional attachment, which is in line with the original expectations. H6a was, therefore, supported. Similarly, the moderating effect of self-perception of ageing on the perceived system quality and emotional attachment as well as the perceived service quality and emotional attachment was verified, supporting H6.

Note that we used sex, age, and education level as control variables; the T-test results showed that sex, age, and education level had no significant impact on the willingness of older people to use intelligent aged-care products, which means that the attribute variables had no significant impact on the results of the empirical analysis. The final hypothesis test results are shown in Table 6.

We found that all the other hypotheses were supported except for that regarding perceived information quality, which had no significant impact on an older age group’s willingness to use intelligent aged-care products. 

## 5. Discussion

This study is based on the information system success model combined with attachment theory, and we incorporated the variables of the self-perception of ageing into the model to construct a model of the mechanism influencing the willingness of older people to use intelligent aged-care products in aged-care organizations. We conducted field investigations and administered questionnaires and analyzed the correlation of various variables in the theoretical model through structural equation models.

### 5.1. Main Findings

The main results are as follows:

Older people’s perception of the quality of intelligent elderly care service products partially impacts their willingness to use them. The research results showed that older people’s perceived information quality and perceived service quality of intelligent aged-care products significantly increased their willingness to use these products, which is consistent with previous studies [11,12,37,38,39]. Moreover, the perceived service quality had the greatest impact on their willingness to use these products, indicating that older people are more concerned with the service experience perception of the products than other age groups [11]. However, older people’s perceived information quality of intelligent aged-care products had no significant impact on their willingness to use them. This may be due to the limited knowledge and understanding of health data by older people [73], leading to trouble understanding some health data information presented by the aged-care service products. This difficulty understanding the health meaning behind the digital information leads to a lack of significant impact on the willingness to use the products.

Older people’s perception of the quality of intelligent elderly care service products and self-perception of ageing each had a significant positive impact on their emotional attachment. When older people had a high perception of the quality of intelligent aged-care products, it promoted their positive emotional response to the product [42]. For example, when an individual perceives a high-quality product, it is easier to have a feeling of trust in and satisfaction with the product [41], thereby enhancing the emotional attachment of older people to the product. Similarly, a more positive self-perception of ageing encourages individuals to adopt a more active and healthier lifestyle [74], which, in turn, results in stronger emotions toward products that can help them understand their own health status.

The self-perception of ageing of older people and their emotional attachment to intelligent aged-care products each had a significant positive impact on the willingness to use these products. The more positive the self-perception of ageing was, the stronger the health awareness of older people was, and the more active they will be in understanding their own health status [75], which will promote their willingness to use intelligent aged-care products. Similarly, the emotional attachment of older people to intelligent aged-care products will promote the formation of a strong relationship between older individuals and the products [51], which will have a lasting and stable impact on the person [76], thus enhancing the willingness to use the product.

### 5.2. Theoretical Contribution

Firstly, the success model of the information system can be evaluated from multiple dimensions and different levels [11]. Different from previous studies, the object of this study focuses on an older age group, including perceived self-ageing into the model, and discussing older people’s willingness to use pension service products from a technical dimension (information quality, system quality, service quality) and an individual dimension (perceived self-ageing), respectively. To a certain extent, it expands the application background of the information system success model.

Secondly, considering the characteristics of an older age group, compared with other age groups, due to the decline in physical function, older people are more likely to develop an emotional attachment to certain trust items. This is due to certain characteristics common among older people, such as the decline in physical ability or memory loss. The stronger the emotional attachment is, the more motivated they are to invest their personal resources in using the product. Compared with a young age group, the older age group has a greater degree of attachment to intelligent aged-care products. Therefore, we include affective attachment into the model to further improve the application of the information system success model in older people.

Thirdly, based on attachment theory, we included emotional attachment variables in the model, and analyzed the mechanism of the influence of older people’s perceptual factors on the willingness to use intelligent aged-care products. Previously, some scholars showed that a perception of high product quality will produce positive emotional responses [42], but studies on the emotional attachment of older people to intelligent aged-care products are limited. Many previous studies showed that an emotional attachment impacts consumers’ buying intention and behavior in different contexts [51,54,55], willingness to participate [58], and willingness to use [13]. We applied emotional attachment theory to the willingness of older people to use intelligent aged-care products, enriching the application scenarios of emotional attachment theory and having important theoretical research significance.

Finally, through empirical research methods, we constructed a theoretical model of the willingness of older people to use intelligent aged-care products and validated it. Previous studies focused more on the design process of such intelligent aged-care products, with a lack of research on the willingness to use these products based on the perspective of an older age group. Based on the success model of the information system, we combined emotional attachment theory to study the influence mechanism of older people’s willingness to use intelligent aged-care products, enriching the theoretical basis of the willingness to use these products and laying a theoretical foundation for subsequent research.

### 5.3. Practical Significance

With the increase in the number of older people in China, while addressing the insufficient supply of aged-care services, many aged-care organizations are focusing on improving service quality and the levels of intelligence and informatization. The results of this study have important practical significance for improving older people’s health literacy, the intelligence level of aged-care organizations, and their overall service quality.

For older people, the findings provide understanding of the actual needs of older people for intelligent aged-care products and the role played by those products, thereby enhancing older people’s health literacy.

For aged-care organizations, through research on the mechanism influencing older people’s willingness to use intelligent aged-care products, the application of intelligent aged-care services in the organization can be improved. The use of these products can also reduce the pressure on the organization’s nursing staff, also strengthening the competitiveness of the organization.

### 5.4. Limitations and Future Prospects

Firstly, in this study, the data collection was not wide enough, and only involved elderly people in some aged-care organizations in Anhui Province. There may be differences in the application degree of intelligent aged-care products in different aged-care organizations. Therefore, the research was influenced by space and research object constraints. Research can be conducted on elderly groups in aged-care organizations in different regions, and research and data collection can be performed with aged-care organizations of different levels and in subsidized areas to aid in the construction of corresponding theoretical models for research.

Secondly, we constructed a theoretical model of the willingness of older people to use intelligent aged-care products from the perspective of product quality perception and emotional attachment. Research can be conducted from different perspectives. Other influencing factors can be considered from the individual level, emotional attachment can be further divided into dimensions, and social level influencing factors can be added for further research and discussion.

Thirdly, one of the limitations of this study is that it did not take into account the demographic characteristics of people with co-existing diseases. Older adults face challenges related to age and illness that may affect their use of information technology [77]. Older people who are in good health and have high cognitive ability can affect the use of information technology [78]. Future research could explore the demographic characteristics of coexisting diseases as factors in this theoretical model.

## Figures and Tables

**Table 1:** Variables and indicators used in the research.

Latent Variable	Measurement Item	Reference
Perceived information quality (PIQ)	PIQ1 I think the health information in the intelligent aged-care products is updated in time.	[23,60]
	PIQ2 I think the health information in intelligent aged-care products is accurate.	
	PIQ3 I think the health information in intelligent aged-care products is comprehensive.	
Perceived system quality (PSYQ)	PSYQ1 I think intelligent aged-care products are reliable.	[23,60]
	PSYQ2 I think it is efficient for intelligent aged-care products to obtain health information.	
	PSYQ3 I think the use of intelligent aged-care products is effective.	
	PSYQ4 I think the layout of intelligent aged-care products is clear.	
	PSYQ5 I think the operation of intelligent aged-care products is safe.	
Perceived service quality (PSEQ)	PSEQ1 I think intelligent aged-care products can provide reliable services.	[23,60]
	PSEQ2 I think intelligent aged-care products can provide timely services.	
	PSEQ3 I think intelligent aged-care products can provide personalized services.	
Self-perception of ageing (SPA)	SPA1 As I grow older, I feel my condition is getting worse.	[61]
	SPA2 I am as energetic as last year this year.	
	SPA3 As I get older, I feel that people are becoming more and more useless.	
	SPA4 I am as happy now as when I was young.	
Emotional attachment (EA)	EA1 I am emotionally connected with intelligent aged-care products.	[62,63]
	EA2 I am passionate about using intelligent aged-care products.	
	EA3 I am emotionally attached to intelligent aged-care products.	
Willingness to use (WTU)	WTU1 I am willing to use intelligent aged-care products	[64,65]
	WTU2 When using it, I am willing to prioritize intelligent aged-care products.	
	WYU3 I am willing to recommend intelligent aged-care products to friends around me.	
	WTU4 I will continue to use intelligent aged-care products in the future.	
	WTU5 I am not interested in intelligent aged-care products.	

**Table 2:** Sample statistics.

Item	Characteristic	Number	Percent
Sex	Male	121	50.21%
	Female	120	49.79%
Age (years)	55–59	17	7.05%
	61–65	58	24.07%
	66–70	97	40.25%
	>70	69	28.63%
Education	High school/technical secondary school and below	129	53.53%
	Junior college	42	17.43%
	Undergraduate	50	20.75%
	Master’s degree and above	20	8.30%
Marital status	Married	115	47.72%
	Divorced	56	23.24%
	Separated	47	19.50%
	Widowed	23	9.54%
Monthly income (yuan)	Below 2500	33	13.69%
	2501–3500	80	33.20%
	3501–4500	59	24.48%
	4501–5500	39	16.18%
	Above 5501	30	12.45%

**Table 3:** Data descriptive statistics results.

Variable	Variable Item	Mean Value	Standard Deviation	Factor Loading
Perceived information quality	PIQ1	4.647	1.639	0.881
	PIQ2	4.867	1.694	0.903
	PIQ3	4.797	1.789	0.901
Perceived system quality	PSYQ1	4.452	1.556	0.86
	PSYQ2	4.56	1.718	0.901
	PSYQ3	4.577	1.72	0.882
	PSYQ4	4.415	1.536	0.863
	PSYQ5	4.444	1.553	0.853
Perceived service quality	PSEQ1	4.564	1.606	0.915
	PSEQ2	4.548	1.759	0.929
	PSEQ3	4.573	1.753	0.917
Self-perception of ageing	SPA1	4.573	1.674	0.873
	SPA2	4.365	1.682	0.884
	SPA3	4.452	1.491	0.884
	SPA4	4.593	1.599	0.888
Emotional attachment	EA1	4.402	1.672	0.877
	EA2	4.515	1.724	0.895
	EA3	4.382	1.725	0.905
Willingness to use	WTU1	4.465	1.549	0.887
	WTU2	4.602	1.51	0.841
	WTU3	4.544	1.485	0.852
	WTU4	4.768	1.574	0.874
	WTU5	4.66	1.538	0.834

**Table 4:** Reliability and validity test results.

Item	Alpha	CR	AVE	WTU	PIQ	EA	SPA	PSEQ	PSYQ
WTU	0.91	0.933	0.736	0.858					
PIQ	0.876	0.924	0.801	0.627	0.895				
EA	0.872	0.921	0.796	0.783	0.589	0.892			
SPA	0.905	0.934	0.778	0.791	0.655	0.717	0.882		
PSEQ	0.91	0.943	0.847	0.778	0.538	0.716	0.722	0.92	
PSYQ	0.921	0.941	0.76	0.789	0.568	0.727	0.765	0.705	0.872

**Table 5:** Moderating effect test 1.

Variable	Emotional Attachment		Emotional Attachment	
	Standardized Coefficient	*T* Value	Standardized Coefficient	*T* Value
Sex	−0.037	−0.813	−0.038	−0.844
Age	0.026	0.561	0.031	0.667
Educational background	−0.052	−1.142	−0.067	−1.488
Perceived information quality	0.21 **	3.583	0.143 *	2.291
Self-perception of ageing	0.573 **	9.684	0.545 **	9.205
Perceived information quality and Self-perception of ageing			−0.15 **	−2.8
R^2^	0.532		0.545	
*F* value	55.47		48.876	

Note: * *p* < 0.05; ** *p* < 0.01.

**Table 6:** Structural parameter estimation results.

Hypothetical Path	Path Coefficient	T Statistics	*p* Statistics	Conclusion
H1a: Perceived information quality → willingness to use	0.086	1.945	0.052	Not supported
H1b: Perceived system quality → willingness to use	0.236 **	3.474	0.001	Supported
H1c: Perceived service quality → willingness to use	0.248 **	5.037	0	Supported
H2a: Perceived information quality → emotional attachment	0.14 *	2.244	0.025	Supported
H2b: Perceived system quality → emotional attachment	0.297 **	4.090	0	Supported
H2c: Perceived service quality → emotional attachment	0.300 **	4.540	0	Supported
H3: Self-perception of ageing → emotional attachment	0.183 *	2.408	0.016	Supported
H4: Self-perception of ageing → willingness to use	0.207 **	3.983	0	Supported
H5: Emotional attachment → willingness to use	0.234 **	5.108	0	Supported
H6: The moderating effect of self-perceived ageing on perceived product quality and emotional attachment	-	-	-	Supported

Note: * *p* < 0.05, ** *p* < 0.01.

## Data Availability

The data presented in this study are available upon reasonable request from the author.
